# Effects of Natural Ventilation and Saliva Standard Ejectors during the COVID-19 Pandemic: A Quantitative Analysis of Aerosol Produced during Dental Procedures

**DOI:** 10.3390/ijerph18147472

**Published:** 2021-07-13

**Authors:** Imena Rexhepi, Rocco Mangifesta, Manlio Santilli, Silvia Guri, Piero Di Carlo, Gianmaria D’Addazio, Sergio Caputi, Bruna Sinjari

**Affiliations:** 1 Unit of Prosthodontics, Dental Clinic, Department of Innovative Technologies in Medicine and Dentistry, University “G. d’Annunzio” of Chieti-Pescara, 66100 Chieti, Italy; imena.rexhepi@unich.it (I.R.); santilliman@gmail.com (M.S.); gianmariad@gmail.com (G.D.); scaputi@unich.it (S.C.); 2 Occupational Allergy, Nanomaterials and Fibers Biosafety and Immunotoxicology Group, CAST, University “G. d’Annunzio” of Chieti-Pescara, 66100 Chieti, Italy; r.mangifesta@unich.it; 3 Durres Regional Hospital, Rruga Telat Noga, 2001 Durrës, Albania; silvaguri@hotmail.com; 4 Department of Innovative Technologies in Medicine and Dentistry, University “G. d’Annunzio” of Chieti-Pescara, 66100 Chieti, Italy; piero.dicarlo@unich.it

**Keywords:** aerosols, COVID-19, dentistry, SARS-CoV-2, standard saliva ejectors, dental infection control

## Abstract

The novel Coronavirus Disease 2019 (COVID-19) pandemic has renewed attention to aerosol-generating procedures (AGPs). Dental-care workers are at high risk of contamination by SARS-CoV-2. The aim of this study was to evaluate the efficacy of standard saliva ejectors and natural ventilation in reducing particulate matter (PM) concentration during different routine dental procedures in the pandemic period. The DustTrak monitor was used to measure PM1, PM2.5, PM10, and breathable (<4 microns) total dust during 14 procedures performed with and without the presence of natural ventilation in a dental unit. Moreover, measurements were performed near the practitioners or near the standard saliva ejectors during the different procedures. In the latter condition, reduced levels of PM10 were recorded (82.40 ± 9.65 μg/m^3^ vs. 50.52 ± 0.23 μg/m^3^). Moreover, higher levels of PM (53.95 ± 2.29 μg/m^3^ vs. 27.85 ± 0.14 μg/m^3^) were produced when the dental unit’s windows were open. At the same time, the total level of PM were higher during scaling than during other procedures (data suggest not to adopt natural ventilation—both window and door opened—during dental procedures). It was also demonstrated that the use of standard saliva ejectors can considerably reduce the total released amount of PM10.

## 1. Introduction

The ongoing novel coronavirus disease (COVID-19) represents the most significant public health emergency that the world has faced in the last century, with over 114 million confirmed cases and 3,823,206 reported deaths by 15 June 2021 [1]. SARS-CoV-2 is transmitted primarily through direct or indirect contact with infected droplets. However, transmission of SARS-CoV-2 through aerosol has been proved, particularly in the presence of highly concentrated aerosols in closed environments [2]. Evaluations of the air and surfaces in indoor environments [3] such as restaurants, post offices, pharmacies or in more confined environments such as city buses [4] are pivotal to better understand SARS-CoV-2 spreading and airborne transmission. In fact, findings suggest that air ventilation and wearing facial masks can further mitigate virus transmission among people [5]. Aerosol-generating procedures (AGPs) in dental practice remain a health concern, since aerosols produced during clinical procedures can be contaminated with microorganisms, which can cause respiratory health effects or transmit diseases bidirectionally among dental professionals and patients [6]. In fact, AGPs can allow pathogens, such as Sars-Cov-2, to spread over considerable distances and also remain suspended in the air for several hours, making the working environment an area with a high risk of nosocomial spread [7]. According to their size, aerosol particles can be classified in ultrafine particles (less than 1 micron in size, PM1 particulate matter classification), fine particles (less than 2.5 microns, PM2.5), and coarse particles (2.5–10 microns, PM10) [8]. The oral–nasal airway can allow the flow of air particles larger than PM10. This poses a risk, as PM10 is able to enter the respiratory system, and both PM2.5 and PM1 can enter the alveolar sac. PM10 level is widely adopted as an indoor air quality indicator. In fact, the presence of several pathogens in PM, including viral particles (0.1%, in both PM10 and PM2.5), has been detected [9,10]. Moreover, prolonged exposure to high PM2.5 and PM10 concentrations can stimulate the overexpression of alveolar ACE-2 receptors, which are responsible for the binding of Sars-Cov-2 in human tissues [11]. In dental settings, it was demonstrated that fluorescein introduced into irrigation systems could be found as far as 4 m from the saliva ejectors during high-speed dental handpieces use [12]. With respect to the Sars-Cov-2 pandemic, the Guidance for Dental Settings issued by the American federal agency of the CDC (Center of Disease Control and Prevention) recommends to dental practitioners to “avoid aerosol-generating procedures whenever possible” and, if it is not possible, to adopt several protective measures regarding AGPs, including natural ventilation by opening dental units’ windows so to reduce the transmission of highly virulent pathogens [13]. The importance of frequently renewing the air after each procedure performed in a dental office was also reported [14]. To date, studies have assessed the size and concentration of particles produced by dental practitioners during dental procedures such as tooth drilling [15,16]. It was reported that dental procedures produced considerably more PM1 than particles suspended in the background air [16]. Other findings reported higher PM2.5 and PM10 concentrations during dental procedures than during non-working periods (i.e., indoor background) [16,17,18]. In this context, the efficacy of mitigating measures such as suction and ventilation in reducing the potential viral load of aerosol and splatter created during dental procedures is not well explored [19]. Although previous studies focused on how dental treatments release a large amount of bioaerosol, there is currently a great heterogeneity regarding the methodologies applied in order to quantify air contamination in dental environments. Moreover, there is a lack of studies evaluating bioaerosol profiles produced under different working conditions [20,21]. Therefore, the aim of this study was to investigate the spreading of PM concentration in aerosol fractions released during different dental treatments in the presence of natural ventilation. Moreover, the effectiveness of standard saliva ejectors in reducing PM concentration produced in a dental unit was also investigated. The null hypothesis was that no differences existed for particle concentration with or without the presence of natural ventilation during the same dental procedure.

## 2. Materials and Methods

### 2.1. Study Design

An observational study was carried on the first dental activities performed after the lockdown period according to the guidelines available in Italy [22]. A dental unit located in an open plan clinic of the Department of Innovative Technologies in Medicine and Dentistry of the University “G. d’Annunzio” of Chieti-Pescara, Italy, was selected for this study.

The measurements in the dental office were conducted during a three-week period in September 2020. During this period, dental activities were limited in order to organize patient flux, following the indications of the Ministry of Health [22].

Dental activities such as professional oral hygiene practices, conservative dental therapy, prosthetic reconstruction, dentoalveolar surgery, and implant surgery were included for the study evaluations. Specifically, restorative treatments were carried out using rubber dam as the method of isolation of the operative field, dental extractions involved tooth sectioning without performing soft tissue incisions and ostectomies, and implant surgery was performed without any bone regeneration intervention. Tooth preparations for fixed prostheses were carried out using the high-speed high-torque handpiece of the unit chair.

Fourteen different dental procedures were performed with the DustTrak DRX Aerosol Monitor Model 8534 (DustTrak TSI Incorporated, Shoreview, MN, USA) [23]. This real-time monitor conforms with ISO 21501-4 and is accurate to within ±95% with a 5% particle coincidence loss. The sampler software allows data to be analyzed, synthesized, and graphed. The goals of this study were, therefore, to investigate:

- The effects of natural ventilation on the reduction of PM in the dental unit
- The effectiveness of low-volume suction (40 L/min air) in reducing PM concentrations during dental procedures
- The difference in terms of PM between the scaling procedures and the other procedures performed. This discriminant was selected following previous literature and guidelines that described ultrasonic scaling as the dental activity that produces the greatest amount of airborne contamination [24].

### 2.2. Study Setting

Different dental practitioners at the Department of Innovative Technologies in Medicine and Dentistry performed the dental procedures included in the study using the same chair unit. The room’s dimensions were 2.8 m × 2.8 m × 3 m (W × L × H). All practitioners used a protective equipment during the procedures, including a face mask (filtering facepiece level 2), gloves, a face shield, goggles and protective outerwear such as disposable gown.

In order to avoid external factors affecting the results, in the operating room, air conditioning was switched off, and the unit employed the normal equipment functionalities (for example, water cooling and standard saliva ejector) on the chair unit used for daily practice [20]. A real-time monitor was used to measure particulate matter (PM) mass fractions for each particle size every 2 s during the procedure analyzed, and the measurement duration was 90 min for each procedure, consisting of continuous slots including leaving the room in a standstill condition with no operation, performing the dental procedure, and cleaning and sanitizing the operating room. Thus, 15,574 values were recorded in each session. Each test lasted an average of 40 min. Each test therefore acquired about 1112 values.

### 2.3. Working Distance

**Figure 1:**
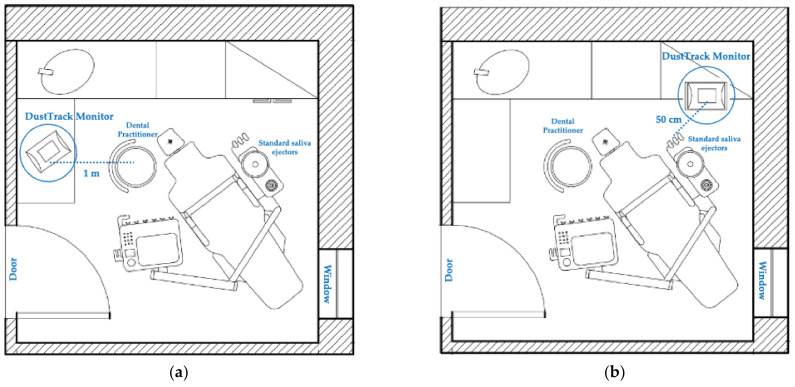
Position of the DustTrak DRX Aerosol Monitor in the plan of the dental unit in which the dental services were performed: (**a**) 1 m from the dental practitioner chair; (**b**) 50 cm from the standard saliva ejectors.

Seven dental activities were performed by positioning the real-time monitor at a distance of 1 m (position A) from the position of the dentists involved in the study, as shown in Figure 1a. The other seven procedures were carried out by positioning the real-time monitor at a distance closer to the aerosol source (50 cm) (position B), near the EM15 saliva ejector (Monoart^®^ Euronda, Vicenza, Italy), as also shown in Figure 1b.

### 2.4. Validation and Background Level

A real-time monitor was exploited to detect the mass of particles in fractions of various size (range of 0.1–10 µm) and their total amount during the procedures performed in order to analyze the current particulate matter in the operating room. The ranges were: PM1: 3 –6μg/m^3^, PM2: 5–68 μg/m^3^, and PM10: 710 μg/m^3^.

### 2.5. Statistical Analyses

Statistical analysis was performed using SPSS software 11.0 (SPPS Inc, Chicago, IL, USA), and correlated analysis was applied to PM1, PM2.5, PM10, breathable, and total dust. The variables are reported as media, standard error, minimum, and maximum. The statistical analysis was performed by the parametric test, due to the high number of recorded measurements (15,574). The Student’s *t*-test was used for unpaired data to compare quantitative variables in the two groups. The statistical significance of the differences between the groups was evaluated at an Alpha Level of 0.001.

## 3. Results

### 3.1. Variations in Ventilation

Data related to the natural ventilation of the dental unit showed how with an open window, the amount of PM recorded in the operating room was significantly higher than when the dental unit’s window was closed, as shown in Table 1.

A second analysis was performed by checking the impact of ventilation provided by opening the door and window.

The amount of PM produced during dental procedures performed while both dental unit window and door were open was statistically significantly higher compared to the total PM recorded when the unit window was open and the door was closed, as shown in Table 2.

### 3.2. Effectiveness of the Suction System

The results regarding the effectiveness of the suction system showed a statistically significant reduction of PM10 levels when the real-time monitor was positioned near the standard saliva ejectors. Thus, low-volume suction seemed to have a significant efficacy in reducing PM10 and total particles, while it showed lower effectiveness in reducing ultrafine PM, with a decrease of about 23% for PM1 and no statistically significant differences, as shown in Table 3.

### 3.3. Differences between Dental Procedures

**Figure 2:**
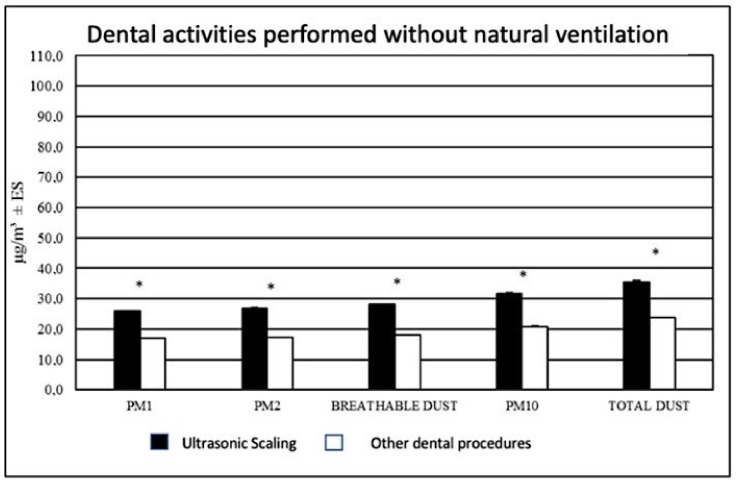
Distribution of aerosol fractions concentration (µg/m^3^) during ultrasonic scaling and other dental activities without natural ventilation. * *p* < 0.001.

Data regarding the levels of PM released during the different dental procedures were also evaluated. It was found that without the presence of natural ventilation (doors and windows closed), a different amount of PM was detected during the different procedures. Specifically, higher levels of PM were found during scaling procedures than during other dental activities, as shown in Figure 2.

## 4. Discussion

The null hypothesis under investigations was rejected. In fact, the results demonstrated that air exchange provided by the opening of the dental unit’s window and door led to higher levels of PM produced during dental procedures. The present study demonstrated that the total concentration of PM produced during the dental procedures can be influenced by several factors in clinical daily practice, such as ventilation, type of procedure, or the use of saliva standard ejectors. Our data provide information on the aerodynamic characteristics of PM release, as well as indications that might be followed during daily dental practice. Moreover, an adequate suction system can considerably reduce PM10 aerosol distribution during dental procedures. In fact, saliva standard ejectors showed a good efficacy in reducing the amount of PM10 particles and total dust produced, but the device seemed less efficient in the aspiration of ultrafine particles, with a reduction of 23% of PM1 and of 50% for total dust, with no statistically significant difference, as shown in Table 3. These data may be related to the mechanisms underlying aerosol diffusion, which include inertial impact, gravitational sedimentation, and Brownian diffusion. Inertial impact occurs between PM10 particles which can be detected by suction systems present in a dental unit. Gravity sedimentation and Brownian motion regard mainly PM2.5 and PM1 particles. In fact, gravity and sedimentation cause the particles to stick to the walls of the upper airways and, when the velocity of the air flow is low, random collisions occur between the particles, transforming them into alveolar sedimentation [25]. Ahmed and Jouhar, in 2021, evaluated the areas of maximum aerosol diffusion during cavity preparation, demonstrating that greater quantities of aerosols are produced in the operator area than in the assistant area [26]. Based on these findings, the use of standard saliva ejectors can significantly reduce the spread of bioareosols produced in the dental field. According to previous findings, a comparison of different suction flow rates indicated that low-volume suction (40 L/min air), like the one used in our study, has a substantial positive effect on contamination in an open clinical environment with partition walls that do not reach the ceiling [27]. In order to have continuous air exchange, an operative air suction could be associated with medical devices for controlled mechanical ventilation, such as HVE [28,29] and HEPA [30,31] filters. HVE filters can effectively reduce the contamination in the operating site by 90% [28]; however, in the absence of a dental assistant, clinicians might find it difficult to operate using one hand [29]. The disadvantages of HEPA filters include the risk that the filter itself can become a source of pathogens, the difficulty in cleaning them, and their high cost [30,31]. It was reported that these devices could be effective in dental offices without windows [28,29,30,31]. In this context, our results showed that natural ventilation increases the amount of PM if the dental unit door is also kept open during dental procedures, as shown in Figure 2, probably due to the aerodynamic mechanism of aerosol diffusion mentioned above. These findings deserve attention in the light of the COVID-19 recommendations provided in order to reduce environmental contamination. In fact, the outbreak of COVID-19, whose symptoms also include oral-related manifestations [32], has clearly placed health professionals at risk of contagion. In this regard, governments around the world have dedicated renewed attention to APGs used in dental daily practice [33]. In fact, governments—including the Italian one—have promoted rules or recommendations to suspend routine dental treatments during the pandemic period [34,35,36]; subsequently, measures to reduce or avoid the production of droplets and aerosols have been strongly recommended, such as not to reduce the fallow time below 10 min in order to prevent larger droplets from settling on exposed surfaces [27,37]. On the same basis, natural ventilation may be recommended before and after each patient’s appointment within the fallow period, in order to provide air ventilation in the operating room. In this case, considerations need to be given to the impact on air temperature of natural ventilation within the operating room, which may depend on several factors such as the degree of thermal discomfort for both dentists and patients and the level of air displacement and ventilation necessary for the dilution of bacterial concentrations [38,39]. Moreover, wind speed and direction and air pollution could be other factors that could interfere with indoor environments [39].

Additionally, for the dental procedures performed, our data showed that without natural ventilation, scaling procedures produced higher PM particle levels compared to other monitored dental activities, in agreement with what was previously reported in the literature [20]. The condition of natural ventilation of a unit might be affected by wind speed and direction, and its effect on the modulation of the amount of PM could be unpredictable, especially in a setting in which the unit door is open. In fact, in order to reduce the risk of infection for dental professionals during the outbreak of COVID-19, it is strongly advised, where possible, to avoid using handpieces and ultrasonic instruments, which are pivotal during periodontal and peri-implant scaling sessions [40].

Otherwise, no differences have been proven in the effectiveness of mechanical over manual instrumentation in non-surgical periodontal procedures, and therefore, it is recommendable to use manual instruments [29,41].

Previous findings reported that higher levels of oral microorganisms were generated during scaling, but over a period of 10–30 min, other aerosol levels returned to baseline [42,43].

Moreover, it was shown, in agreement with our data, that the values of PM1 and PM2.5 did not vary significantly immediately after the initial measurements, since it is assumed that PM10 is heavier and therefore would have precipitate more extensively than PM1 and PM2.5 [44].

The limitations of this study concern several aspects. First, the sample size of dental procedures performed in our study could be increased. In addition, our results on natural ventilation should be interpreted keeping in mind that the air turbulence could affect the levels of PM and that the assessments performed did not concern the other procedures carried out in the adjacent bays of the open plan clinic setting. Further studies are necessary to better understand the present results, probably using other instrumentations and other conditions and monitoring the level of outdoor PM.

## 5. Conclusions

In regard to the Sars-Cov-2 pandemic, the international guidelines [13] recommend to dental practitioners to adopt protective measures including natural ventilation and to avoid APGs whenever possible to reduce the risk of cross infections. In relation to these recommendations, our results suggest that natural ventilation may not be recommendable during the performance of dental activities. In fact, high levels of PM were recorded in the presence of natural ventilation, and it is advisable, if no mechanical ventilation is available, not to leave the door of a dental unit open during the procedures performed in an open plan clinic setting. Moreover, low-volume suction can considerably reduce the total amount of PM10 produced in the dental unit also during dental activities such as ultrasonic scaling, which seems to release, under standard conditions, high levels of PM, as also previously reported. Thus, as suggested by the current guidelines [13], the use of airborne spreading devices should be minimized in order to reduce the production of PM particles that can act as vectors of infectious agents and contaminate dental practitioners, patients, and working surfaces in the operating space.

## Figures and Tables

**Table 1:** Distribution of aerosol fractions concentration (µg/m^3^) with and without natural ventilation (dental unit window open). A statistically significant difference was shown between the amounts of PM produced in the two conditions. All experiments were conducted with the unit door closed.

Parameter	Real Time Monitor		*p*-Value ^a^
	Dental Unit Window Open Mean ± SE (µg/m^3^)	Dental Unit Window Closed Mean ± SE (µg/m^3^)	
PM_1_	34.49 ± 0.72	20.73 ± 0.08	<0.001 *
PM_2.5_	35.34 ± 0.76	21.26 ± 0.08	<0.001 *
BREATHABLE DUST	37.1 ± 0.86	22.14 ± 0.09	<0.001 *
PM_10_	44.66 ± 1.45	25.01 ± 0.1	<0.001 *
TOTAL DUST	53.95 ± 2.29	27.85 ± 0.14	<0.001 *

^a^ Student’s *t*-test, * *p* < 0.001.

**Table 2:** Distribution of aerosol fractions concentration (µg/m^3^) recorded while the dental unit door was open. A statistically significant difference was shown between the amounts of PM produced in the two conditions.

Parameter	Real Time Monitor		*p*-Value ^a^
	Dental Unit Door Closed Mean ± SE (µg/m^3^)	Dental Unit Door Open Mean ± SE (µg/m^3^)	
PM_1_	34.49 ± 0.72	45.98 ± 0.1	<0.001 *
PM_2.5_	35.34 ± 0.76	47.15 ± 0.12	<0.001 *
BREATHABLE DUST	37.1 ± 0.86	50.34 ± 0.14	<0.001 *
PM_10_	44.66 ± 1.45	64.32 ± 0.09	<0.001 *
TOTAL DUST	53.95 ± 2.29	82.37 ± 0.12	<0.001 *

^a^ Student’s *t*-test, * *p* < 0.001.

**Table 3:** Distribution of aerosol fractions concentration (µg/m^3^) during different dental procedures when the DustTrak DRX Aerosol Monitor was positioned near the saliva standard ejectors.

Parameter	Real Time Monitor		*p*-Value ^a^	% ∆
	Position A (Near Practitioner Chair) Mean ± SE (µg/m^3^)	Position B (Near Standard Saliva Ejectors) Mean ± SE (µg/m^3^)		
PM_1_	52.51 ± 4.81	40.71 ± 0.16	0.007	−22.6
PM_2.5_	54.34 ± 5.07	41.44 ± 0.16	0.005	−23.7
BREATHABLE DUST	58.49 ± 5.70	43.37 ± 0.16	0.004	−25.8
PM_10_	82.40 ± 9.65	50.52 ± 0.23	<0.001 *	−38.7
TOTAL DUST	117.17 ± 15.20	58.54 ± 0.38	<0.001 *	−50.5

^a^ Student’s *t*-test, * *p* < 0.001.

## Data Availability

The data that support the findings of this study are available from the corresponding author, upon reasonable request.
